# Polyamide/Poly(Amino Acid) Polymers for Drug Delivery

**DOI:** 10.3390/jfb12040058

**Published:** 2021-10-08

**Authors:** Sai H. S. Boddu, Prakash Bhagav, Pradeep K. Karla, Shery Jacob, Mansi D. Adatiya, Tejas M. Dhameliya, Ketan M. Ranch, Amit K. Tiwari

**Affiliations:** 1 Department of Pharmaceutical Sciences, College of Pharmacy and Health Sciences, Ajman University, Ajman P.O. Box 346, United Arab Emirates; 2 Center of Medical and Bio-Allied Health Sciences Research, Ajman University, Ajman P.O. Box 346, United Arab Emirates; amit.tiwari@utoledo.edu; 3 Advanced Drug Delivery Research and Development, Sampann Research and Development, Panacea Biotec Ltd., Ambala, Chandigarh Highway, Lalru 140501, India; prakashbhagav@gmail.com; 4 Department of Pharmaceutical Sciences, College of Pharmacy, Howard University, 2300 4th St. N.W., Washington, DC 20059, USA; 5 Department of Pharmaceutical Sciences, College of Pharmacy, Gulf Medical University, Ajman 4184, United Arab Emirates; sheryjacob6876@gmail.com; 6 Lallubhai Motilal College of Pharmacy, Navrangpura, Ahmedabad 380009, India; mansi19adatiya@gmail.com (M.D.A.); tejas.dhameliya@lmcp.ac.in (T.M.D.); ranchketan@gmail.com (K.M.R.); 7 Department of Pharmacology & Experimental Therapeutics, Health Science Campus, The University of Toledo, 3000 Arlington Ave., Toledo, OH 43614, USA

**Keywords:** polyamides, poly(amino acid)s, drug delivery, micelles, nanocarriers, biopolymers

## Abstract

Polymers have always played a critical role in the development of novel drug delivery systems by providing the sustained, controlled and targeted release of both hydrophobic and hydrophilic drugs. Among the different polymers, polyamides or poly(amino acid)s exhibit distinct features such as good biocompatibility, slow degradability and flexible physicochemical modification. The degradation rates of poly(amino acid)s are influenced by the hydrophilicity of the amino acids that make up the polymer. Poly(amino acid)s are extensively used in the formulation of chemotherapeutics to achieve selective delivery for an appropriate duration of time in order to lessen the drug-related side effects and increase the anti-tumor efficacy. This review highlights various poly(amino acid) polymers used in drug delivery along with new developments in their utility. A thorough discussion on anticancer agents incorporated into poly(amino acid) micellar systems that are under clinical evaluation is included.

## 1. Introduction

Novel polymer-based drug delivery systems are being designed in order to alter the physicochemical and pharmacokinetic properties of drug molecules and to deliver them effectively in a controlled manner. Polymers have garnered attention in the evolution of drug delivery technologies for both water-soluble and hydrophobic drug molecules [1]. The alteration and application of various polymeric materials unquestionably proved to be useful in treating diseases where conventional therapies failed to achieve the required bioavailabilities. Though it is difficult to synthesize drugs with ideal physicochemical and pharmacological properties, shrewd manipulation of delivery systems with polymers can make the final formulation ideal for a particular disease condition. However, these novel polymers must be accepted by the Food and Drug Administration (FDA) before they are used in the delivery of drugs. During the last two decades, formulations like time-release medications that regulate the rate/period of drug release have become common. With the detailed understanding of the human body, it is now feasible to introduce novel treatment options with high patient compliance [2]. 

In the past, active ingredients were identified and extracted from plant material for the treatment of diseases. However, with the advancements in synthetic chemistry, new drugs for treatment of a particular disease are being introduced through high-throughput screening (HTS). Using this technique, it is possible to test huge libraries of chemicals for their ability to treat the disease. Nonetheless, the full potential of drug molecules can only be accomplished with well-designed delivery systems. The chief hurdle confronting the drug delivery scientists is the design of novel ways to deliver active pharmaceutical ingredients to the target site. Developments in polymer chemistry created new opportunities to deliver drugs near to the target region in a required fashion. At the beginning of 1970s, polymers composed of lactic acid were first employed for drug delivery. The release of the drug from polymers occurs in a cyclic fashion and the release can be further triggered by ambient conditions near the target site. Polymers utilized in drug delivery should bioresorbable, biocompatible and degrade in the body [3]. Nevertheless, certain biocompatible and non-biodegradable polymers (e.g., ethyl-vinyl acetate (EVA)) are also being used to sustain the duration of release for more than one year. Based on the source, polymers can be classified into: (A) natural polymers [examples: proteins (gelatin, casein, silk and wool), polysaccharides (starch, cellulose), polyesters (polyhydroxyalkanoates), and others (natural rubber, lignin and shellac)]; and (B) synthetic polymers [examples: poly ethylene, poly urethane, poly siloxanes, polyalkylene esters, polyamides, polyacrylamide, poly(2-hydroxy ethyl methacrylate), polyamide esters, poly(methacrylic acid), polyvinyl esters, poly(N-vinyl pyrrolidone), polyvinyl alcohols, poly lactic acid and its co-polymers, polydioxanone, poly(ethylene glycol), poly(bisphenol A iminocarbonate), poly(acrylic acid), poly(methyl methacrylate), poly(ethylene-co-vinyl acetate) and polyanhydrides] (Table 1). 

**Figure 1:**
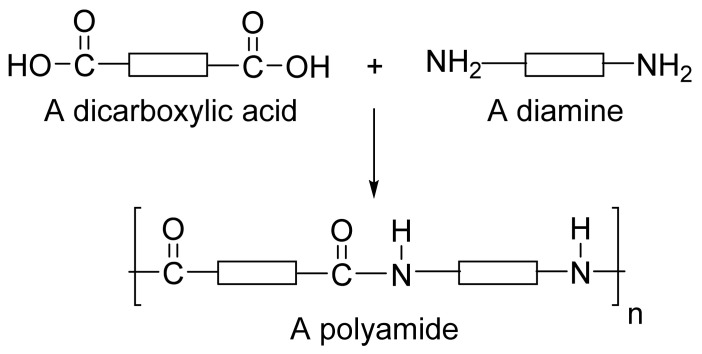
Structure of a polyamide.

Polyamide is a polymer with repeating units linked by amide bonds, while a group of small polyamides containing multiple amino acids of the same types linked through amide bonds is often referred to as poly(amino acid)s. Polyamide occurs naturally or can be made synthetically (Figure 1) [4]. Naturally occurring polyamides include proteins, such as silk and wool, while synthetically made polyamides include materials such as nylons, sodium poly(aspartate) and aramids [5]. Polyamides can also be categorized into: homopolyamides with one kind of monomer, and copolyamides with different constituents [6]. Polyamide was first introduced as the toothbrush filaments of polyamide 6,6 by DuPont in 1938. By 1950, it found applications in the plastic industry due to its attractive properties such as thermal stability, good chemical resistance, relatively high tensile strength and stiffness [7]. Polyamides possess excellent mechanical characteristics, good sliding and wearing characteristics. Polyamides are distinguishable based on mechanical properties PA 66 (hard and tough) and PA 12 (soft with flexible properties). Marketed types of polyamides include polyamide 4,6; polyamide 6; polyamide 6,6; polyamide 6,12; polyamide 11; and polyamide 12 amongst which most of them are used as thermoplastics. In the 2000s, polyamide polymers were used to incorporate water-insoluble and metabolically unstable anti-neoplastic drugs. The advantages of using polyamide polymers as the drug delivery system include localized target site action, sustained release and stabilization. However, microbial contamination, excessive hydration and reduced viscosity on storage are disadvantages [8]. Aliphatic polyamides complexed with nanoparticles have been explored for multifunctional applications [7]. 

## 2. Polyamide Polymers Used in Drug Delivery

Polymers based on amino acid monomers have gained significant attention in drug delivery. Natural amide-based polymers such as gelatin and albumin are widely used in the preparation of biodegradable microcapsules and microspheres [9]. Synthetic poly(amino acid)s are also investigated in biomedical/drug delivery applications as they are structurally similar with naturally occurring proteins. In general, the application of water-soluble polymer conjugates in drug delivery via carrier-based systems offers several advantages such as improved drug pharmacokinetics, minimal toxicity to vital organs, tissues or cells and enhanced drug buildup at the target site. Moreover, the release of the drug from a carrier at the target tissues can be programmed to achieve a predetermined drug delivery [10]. For instance, in a recent study temperature and pH-responsive pentablock copolymers were synthesized using pH-responsive polyamide as well as temperature-sensitive poly(ε-caprolactone)-b-poly(ethylene glycol)-b-poly(ε-caprolactone) (PCL-b-PEG-b-PCL) for use in controlled drug delivery. The pentablock copolymer was easily soluble at 20 wt.% and resulted in a stable hydrogel at pH 7.4 and a temperature of 37 °C (physiological region). In addition, the sol-to-gel transition of the pentablock copolymers could be changed through altering the polyamide to PCL/PEG ratios [11]. Conjugation of a therapeutic agent to the polymer results in a drug–polymer conjugate with enhanced pharmacokinetic/pharmacodynamic properties such as increased plasma half-life due to prolonged plasma circulation, protection of drugs from proteolytic enzymes, decreased immunogenic response, enhanced stability (proteins and peptides), enhanced solubility of low molecular weight drugs and the possibility for targeted delivery. For example, paclitaxel poliglumex (CT-2103; XYOTAX) synthesized by conjugating paclitaxel and poly-L-glutamic acid. CT-2103 showed higher paclitaxel accumulation in tumor tissues due to its enhanced permeation and retention effect. CT-2103 minimized the systemic exposure and increased the therapeutic index of the drug [12].

**Figure 2:**
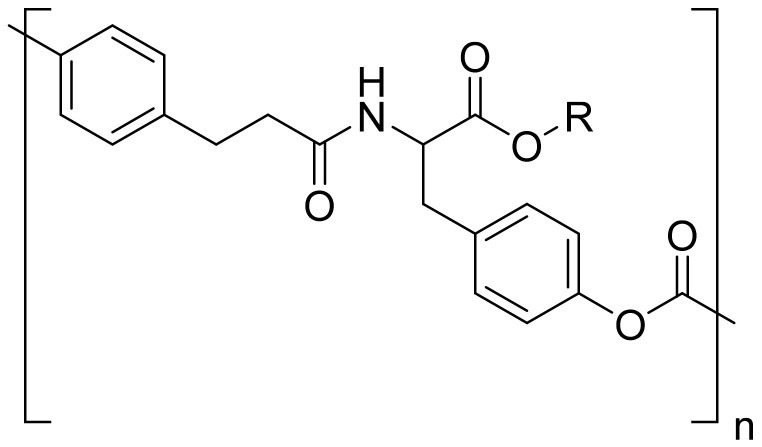
Structure of tyrosine conjugated to polycarbonate.

Amino acids can be grafted on to polymers or copolymerized with other monomers resulting in derived polymers or pseudo-poly(amino acid)s. For example, block copolymers were synthesized by the linking of amino acid sequences with poly(ethylene glycol) using a non-amide bond such as carbonate, ester or iminocarbonate bonds [13,14]. Similarly, tyrosine-derived pseudo-poly(amino acid)s were synthesized by polymerizing tyrosine with phosgene or bis(chloromethyl) carbonate triphosgene (Figure 2). A varying pendant alkyl ester chain results in a polymer with altered physicochemical properties. These are amorphous polymers (T_g_ < 100 °C) with a very low water solubility, and hydrolysis of the polymer results in tyrosine and diols with excellent tissue compatibility [15]. Due to its unique properties, tyrosine-derived polycarbonate is widely investigated for the delivery of bone morphogenetic protein (rhBMP-2) that stimulates bone growth and healing, even in polytrauma and suboptimal healing conditions such as diabetes [16]. In comparison with copolymers containing two or more amino acids, homopolymers of poly(amino acid)s are preferred due to their non-immunogenic nature, and the extent of immunogenicity varies with polymer composition [17]. 

Poly(amino acid)s are the most frequently utilized polyamides in drug delivery. The ambition of this review is to emphasize some of the most important polyamide homo- and copolymers that are widely used in drug delivery such as poly(amidoamine), polyaspartic acid, polylysine and poly(glutamic acid) (Table 2). The unique characteristics of these poly(amino acid) polymers have motivated scientists to study the usefulness of these polymers for several industrial applications in addition to drug delivery [18]. Low-molecular weight drugs are typically delivered using poly(amino acids), which are cleaved by enzymes into nontoxic substances [19]. Poly(amino acid) polymers degrade inside the body resulting in amino acids that are biologically active and are often used as drugs [19]. The following section highlights a few widely used poly(amino acid) polymers in drug delivery. In addition, poly(amino acid)-based micelles in clinical trials such as NK105 and NC-6004 are thoroughly discussed as case studies.

### 2.1. Poly(Aspartic Acid)

Poly(aspartic acid) is widely used in biomedical applications, especially gene delivery and drug delivery [20], due to its low toxicity profile, non-antigenic, excellent biocompatibility and biodegradability [21,22,23]. Poly(aspartic acid)s like other poly(amino acid)s are extensively used in several areas such as water treatment, paper processing and paint additives. In the field of biomedical sciences, poly(aspartic acid) is employed in the design of dialysis membranes, drug delivery systems, hydrogels, orthopedic implants and artificial skin [24,25]. Poly(aspartic acid) derivatives of varying physicochemical properties are easy to synthesize using simple chemical procedures [26]. Poly(amino acid)s, such as poly(aspartic acid), polylysine and poly(glutamic acid) are synthesized by the polymerization of *N*-carboxyanhydrides of α-amino acids. However, this method is not economical, and the pendant reactive amino acids groups should be protected before polymerization. For more information on the methods of synthesis of poly(aspartic acid) homopolymers and copolymers, readers can go see the review article by Roweton et al. [27]. PEGylation of poly(amino acid)s has been used in the controlled and targeted delivery of anti-tumor agents. PEGylated-poly(amino acid)s form micelles with a hydrophobic core enveloped by groups that are water-loving in nature. The core-shell structure favors the entrapment of hydrophobic drug molecules and protects them from precipitation and protein adsorption in the body. Polymeric micelles of poly(aspartic acid) were prepared from PEG-poly(aspartate) to accomplish the pH-dependent drug release in tumor tissues. A 12 kDa PEG and poly(aspartate) (5, 15 and 35 repeating units) were used in the synthesis of block copolymer. Hydrazide linkers with 4-aminobenzoate (Abz) and glycine (Gly) spacers were connected to PEG-poly(aspartate), followed by doxorubicin conjugation. The synthesized polymer–drug conjugates resulted in micelles of size < 50 nm using a dialysis technique. Doxorubicin release from micelles was found to be controlled and pH-dependent due to the addition of Gly and Abz linkers [28]. In a different study, nanoparticles of chitosan-poly (aspartic acid)-5-fluorouracil were prepared by the process of ionic gelation. Initially, chitosan and 5-fuorouracil were sequentially dissolved in a dilute acetic acid (10 gm/mL) solution at room temperature. This solution was then slowly added to the poly(aspartic acid) solution with continuous stirring resulting in an opalescent suspension. Gluteraldehyde cross-linking nanoparticles were dropped into the filtered suspension and kept under stirring for three hours at 25 °C. The suspension was ultra-centrifuged at 35,000 rpm for 30 min at 25 °C in order to separate the chitosan-poly(aspartic acid)-5-fluorouracil nanoparticles from the aqueous suspension. The tumor inhibition rate of chitosan-polyaspartic acid-5-fluorouracil nanoparticles was found to be much higher than 5-fluorouracil alone in a male BABL/c nude mice model of human gastric carcinoma [29]. Poly(aspartic acid) was also used in the design of microdevices. In a recent study, Zhou et al. reported a chemically powered microdevice made up of a Zn core and a thin intermediate layer of Fe. Doxorubicin was linked to the microdevice with a poly(aspartic acid) linker. This device propelled in the presence of gastric acid while its motion could be guided through the magnetic field. This self-propelled microdevice was found to be biocompatible and biodegradable in the presence of gastric juice or by proteases in the GI tract. Such devices could be used in delivering drugs to the specific regions in the gastrointestinal tract [30]. Poly(aspartic acid) complexed with magnetite nanoparticles (or) as a substrate for inorganic quantum dots was employed in theranostics. Further, the hydrophilic character of poly(aspartic acid) enables the creation of vectors for gene therapy without inducing an undesirable immune response [31]. Poly(aspartic acid)-based hydrogels were also used in drug delivery as they exhibit a response to ionic strength and pH. A higher swelling ratio of poly(aspartic acid) was achieved by reducing ionic strength or increasing pH. The degradation of hydrogels occurs from a few days to a month depending on enzyme concentration and temperature. Further, poly(aspartic acid)-based hydrogels are flexible to modification under mild conditions without any catalyst requirement. This feature makes poly(aspartic acid)-based hydrogels promising over conventional poly(acrylic acid) hydrogels [32]. 

### 2.2. Poly-L-Lysine

Poly-L-lysine (or ε-poly-(L-lysine) is a natural homopolymer of L-lysine that is produced in strains of *Streptomyces albulus*. Poly-L-lysine is water soluble and has been used as a delivery vehicle for small drugs and macromolecules as it is easily degraded by cells. Poly-L-lysine is a cationic, homo-polypeptide consisting of ~25–30 L-lysine residues with a mol. wt. of 4700 Da. The empirical formula of poly-L-lysine is C_180_H_362_N_60_O_31_. Amino groups of ε-poly-L-lysine attain a positive charge in the water and hence it is electrostatically adsorbed to the bacterial cell surface [33]. Poly(L-lysine) was conjugated to asialoorosomucoid for the targeted delivery of the gene to hepatocytes [34]. In addition, several other targeting ligands such as folate [35], transferrin [36] and galactose [37] have been conjugated to poly-L-lysine for targeting tumor cells [38]. Poly-L-lysine served as a model polymer in elucidating the problems confronted by nonviral vectors for gene delivery. Poly-(L-lysine) is a +vely charged polymer and hence it is used in the delivery of -vely charged molecules by ionic interactions. Further, the cationic nature of poly-(L-lysine) promotes selective internalization into tumor cells via electrostatically adsorptive endocytosis. For example, poly-(L-lysine)-poly(ethylene glycol)-folate conjugate and fluorescein isothiocyanate conjugated bovine serum (FITC-BSA) albumin were physically complexed by ionic interactions in an aqueous phase. FITC-BSA has a (-) surface charge at neutral pH. Poly-(L-lysine)-poly(ethylene glycol)-folate and FITC-BSA complexes were formed by simple mixing at room temperature for 15 min. The results from this study indicated a higher FITC-BSA uptake by KB cells when complexed with a poly-(L-lysine)-poly(ethylene glycol)-folate conjugate [39]. 

**Figure 3:**
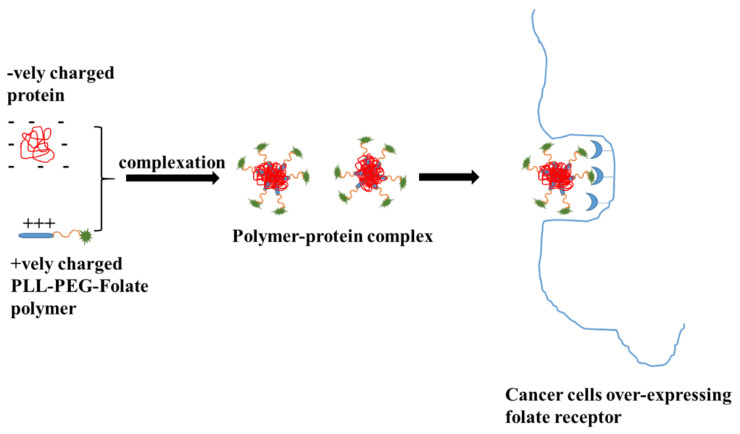
A schematic diagram of folate receptor-mediated intracellular protein delivery using PLL–PEG–FOL conjugate. Modified from Ref. [39].

**Figure 4:**
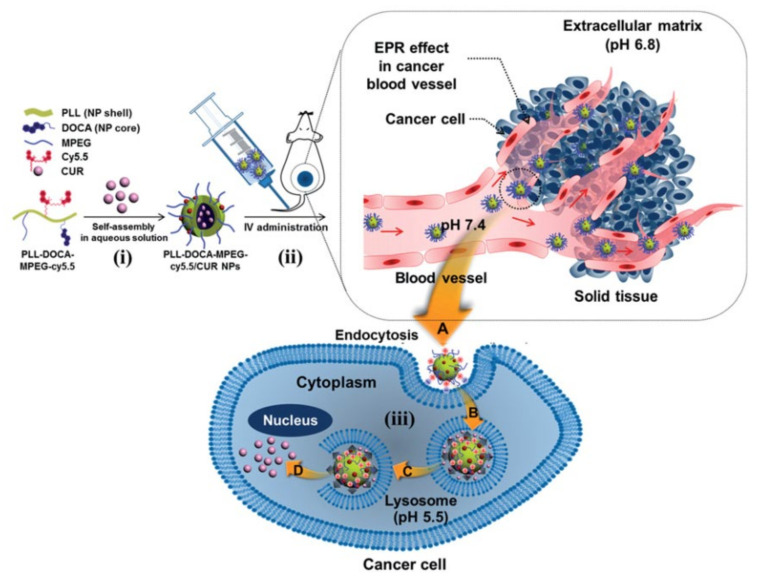
Schematic of the endocytosis of poly-(L-lysine)-deoxycholic acid-methoxy polyethylene glycol-cyanine 5.5/Curcumin nanoparticles into cancer cells and mechanism of the nanoparticles on the apoptosis of the cells. Reproduced with permission from Yang et al. [41]. (i) Self-assembly of poly-(L-lysine)-deoxycholic acid-methoxy polyethylene glycol-cyanine 5.5/Curcumin nanoparticles in aqueous solution (ii) After IV administration NPs circulate through various pH circumstances from veins to the tumor via EPR, and (iii) Endocytosis of poly-(L-lysine)-deoxycholic acid-methoxy polyethylene glycol-cyanine 5.5/Curcumin nanoparticles into cancer cells.

Kim et al. developed a poly-(L-lysine)-poly(ethylene glycol)-folate conjugate basednanoparticulate delivery system for targeting to cancer cells. As shown in Figure 3, in this strategy the +vely charged poly-(L-lysine) anchors on the surface of PLGA nanoparticles −vely charged. Poly-(L-lysine)-poly (ethylene glycol)-folate coated onto PLGA nanoparticles showed a higher uptake in KB cells as compared to unmodified nanoparticles [40]. More recently, a poly-(L-lysine)-based nanoparticulate system was used as a theranostic agent and utilized in the delivery of curcumin (Figure 4). The amine group of poly-(L-lysine) was conjugated to methoxy polyethylene glycol, deoxycholic acid, and cyanine 5.5 through a condensation reaction and further loaded with curcumin using the dialysis method. This study concluded that a poly-(L-lysine)-based nanoparticulate system at low pH showed higher cellular uptake of curcumin into the Hep3B cell line by electrostatically absorptive endocytosis, while the incorporation of cyanine 5.5 into the system provided in vivo and ex vivo biodistribution images [41]. Poly-(L-lysine) is also used as a preservative in food and the pharmaceutical industry due to its ability to kill Gram (+) bacteria and fungi. Poly-(L-lysine) is stable in the presence of heat at 120 °C up to 20 min or 100 °C up to 30 min. The activity of poly-(L-lysine) is reduced in the presence of acidic polysaccharide, chloride, phosphate and copper ions and enhanced with the presence of hydrochloric acid, malic acid, citric acid, glycine and glyceride. Poly-(L-lysine) is widely studied as a nonviral vector for gene delivery as it can condense plasmid DNA to varying degrees based on the salt concentration. Long chain poly-(L-lysine) was found to be efficient in gene delivery, but it is linked to higher toxicity. A linear poly-(L-lysine) without any modification is considered as a poor vector due to low uptake and its inability to undergo an endosomal escape. These are improved by: (a) conjugation to growth factors to enhance the uptake, (b) conjugation to fusogenic peptides to enhance the endosomal escape and (c) PEGylation to extend the t_1/2_ of the complex [42]. Based on the features of poly-(L-lysine) and the success met, it is highly likely that they are attractive next-generation polymers for real-world biomedical applications.

### 2.3. Poly(Glutamic Acid)

Poly(glutamic acid) is an environment-friendly polymer produced by bacterial fermentation [43], especially in Bacillus subtilis and Bacillus licheniformis. It is widely used in controlled and targeted drug delivery. Poly(glutamic acid) is highly soluble in water and hence hydrophobic drugs such as paclitaxel, doxorubicin and daunorubicin are conjugated to poly(glutamic acid) in order to increase its solubility and stability [44,45]. Poly(glutamic acid) is a pseudo-poly(amino acid) polymer with repeat glutamate units. The mol. wt. of poly(glutamic acid) ranges between 100–1000 kDa depending on fermentation time [43]. Poly(glutamic acid) is available in three forms: low molecular weight (200–400 kDa), high molecular weight (800–1000 kDa); and cross-linked poly(glutamic acid) (>10,000 kDa). Poly(glutamic acid) has high water retention properties and it is capable of absorbing 5000 times more water than its own weight. The solubility of poly(glutamic acid) in water reduces with an increase in molecular weight, while the viscosity increases with molecular weight.

Poly(glutamic acid) was used in the preparation of nanoparticles of cisplatin, an anti-cancer drug. A co-ordinate complex was formed between block copolymers of poly(ethylene glycol)-poly(glutamic acid) and cisplatin following incubation for 72 h in distilled water [46,47]. The ligand substitution of platinum (II) atom (Cl^−^ to COO^−^) in the side chain of poly(glutamic acid) is the basis for the formation of cisplatin-incorporated micelles. Such micelles were found to have an enhanced stability compared to the micelles formulated using the amphiphilic block copolymers. This increased stability was attributed to the interpolymer cross-linking by the platinum (II) atom [48]. Block copolymers of γ-poly glutamic acid and poly lactic acid (PLA) were also reported in the literature for drug delivery. The copolymer was synthesized by activating the terminal hydroxyl group of PLA using carbonyldiimidazole (CDI) [49]. The activated PLA and acidified γ-poly glutamic acid were then conjugated to form the block copolymer. Paclitaxel-loaded self-assembling nanocarriers were synthesized using poly(γ-glutamic acid) and poly(lactide). Galactosamine conjugation onto the surface of nanocarriers efficiently reduced the tumor size in hepatoma-tumor-bearing nude mice [50]. Composite biodegradable nanoparticles of chitosan and poly-γ-glutamic acid prepared using the ionotropic gelation process showed higher internalization in A2780/AD ovarian cancer cells that overexpress folate receptors [51]. Similar studies involving chitosan and poly-γ-glutamic acid were reported in the literature [52,53,54,55]. Apart from use in nanocarrier systems, poly-γ-glutamic acid was also used in the preparation of a fully insertable microneedle system. A poly-γ-glutamic acid microneedle with polyvinyl alcohol/polyvinyl pyrrolidone supporting structures delivered insulin in an effective manner in diabetic rats. The hypoglycemic effect produced by the poly-γ-glutamic acid microneedle was comparable to insulin injections administered subcutaneously [56]. In a recent study, composite microparticles of carboxymethyl chitosan and poly-γ-glutamic acid were prepared using an emulsification/internal gelation method. The swelling property of pH sensitive composite microparticles containing levofloxacin released the drug in a sustained manner [57]. During the last decade, poly (glutamic acid) was found to have versatile use in drug delivery and tissue engineering. Currently, this polymer is being studied for its utility in protein and peptide delivery, modulation of the EPR effect and targeted delivery to specific cell types [58].

### 2.4. Poly(Amidoamine)

Synthetic polyamides such as poly(amidoamine) or PAMAM have emerged as a dendritic polymer. PAMAM mimics the protein molecules and has a fairly well-defined topology with various surface functional groups [59]. The peripheral functional groups on dendrimers can be exploited based on their reactivity. Dendrimers are used in several areas including liquid crystals [60], catalysts [61] and photonic devices [62,63,64]. The dendrimer was first introduced into biomedical applications by Tomalia [65] in the 1990s. They mimic biomolecules ranging from simple micelles to complicated highly organized building blocks of biological systems, and hence they are suitable in biomedical applications [66]. Polyamidoamine (PAMAM) dendrimers are perhaps the most common dendrimers with potential biomedical applications. The core of a generation-0 (G-0) PAMAM consists of a diamine or ethylenediamine which is reacted with methyl acrylate, and followed by ethylenediamine to obtain higher generation PAMAMs. Higher generations up to G-10 can be achieved by successive reactions. Lower generations have limited inner space, while medium-sized (G-3 or G-4) have inner space that is detached from the outer dendrimer shell. Very large dendrimers (G-7 and greater) behave like a solid particle with dense surfaces of their outer shell [67]. The molecular weight of the dendrimer approximately doubles with each successive generation. Higher generation dendrimers have more surface functional groups that can be used to customize the dendrimer for a given application [68]. Dendrimers are mainly prepared using the convergent method, divergent method, lego chemistry, click chemistry and self-assembling strategy [69]. In a convergent method, dendrimers are produced by the inward reaction of small molecules, which terminate at the surface of the sphere and subsequently remain attached to the core. While in a divergent method, dendrimers are formed by a chain of outward reactions, which is generally a Michael reaction. Necessary precautions must be taken so that the reaction is complete. This can prevent the production of trialing generations, where all the branches are not equally long. Of the two methods quoted above, dendrimers produced by the convergent method are easy to purify and separate from the shorter branches, resulting in a greater monodisperse dendrimer. In the case of dendrimers produced by the divergent method, purification becomes a problem due to the lesser difference in size between the perfect and imperfect dendrimers. Though the divergent method is used to produce high generation dendrimers due to steric effects [70], it is more labor-consuming and the synthesis process is quite expensive. Click chemistry is also used to synthesize higher generation PAMAM dendrimers within a few days in a reasonable cost-effective manner [71]. Further research is needed into the synthesis strategies of higher generation PAMAM dendrimers.

The following properties of PAMAM dendrimers make them ideal for drug and gene delivery: ability to control the size, biocompatibility, lack of immunogenicity, biodegradability, ability to adhere to cells and permeate the cell membrane via endocytosis, ability to prevent drug degradation and ability to target the drug to specific regions [72,73]. Anticancer drugs could be loaded into the inner void space of dendrimers or linked to the terminal functional groups via covalent bonds or electrostatic interactions. The solubility of hydrophobic drugs could be enhanced using the PAMAM dendrimer and the reactive groups on the dendrimer surface can be modified with ligands for targeting drug delivery. In a recent study, PAMAM dendrimers have been used as a carrier system for reversing the multi-drug resistance (MDR) in human breast cancer cells (MCF-7/ADR cells) [74]. The readers can refer to the recent review article published by Li et al. for detailed information on the use of PAMAM dendrimers in drug and pDNA/siRNA delivery for cancer therapy [72].

### 2.5. Fatty Acid-Based Polyamide

Recently, a fatty acid-based polyamide was reported for drug delivery by Javar et al. [75]. Palmitic acid, a saturated fatty acid, was functionalized with tris(2-aminoethyl)amine, and then polymerized with sebacoyl chloride as a cross-linker. The thin film hydration method was used in the preparation of nanoparticles, in which drug loading was carried out. A large volume of aqueous buffer was added to the resulting thin layer. Later, the hydrophobic polymer was transferred to nanosized particle. These nanosized particles (~80 to 100 nm) play a crucial role in distribution throughout the body. The size of nanoparticles should fall between 10 to 100 nm to produce optimal pharmacokinetic parameters. The smaller size provides a greater surface area of drug exposure to biological fluids, whereas larger particles allow to keep a greater amount of the drug within the core. Nanoparticles of smaller sizes are unable to hold the drug for a longer period which leads to faster degradation. Studies on the surface charge of polymers have shown that nanoparticles with a (+) charge on the surface show better cellular uptake [75].

### 2.6. Others

Polyamide 6,10 is known to provide superior physicochemical and physio-mechanical properties due to its semi crystalline and thermoplastic nature. Owing to its even-even configured linear chain and poly-condensed synthetic aliphatic polymerization, it can be used for absorbable and non-absorbable surgical sutures. Adeleke has reported a synthetic polyamide 6,10 based erodible compact disc. The disc formulation of polyamide 6,10 was found to consistently provide sustained in vitro drug release and erodible tendencies under biorelevant conditions [76]. Montmorillonite (MMT) are naturally occurring inorganic cationic exchangers that works by exchanging ions with the drug solution. MMT plays an important role in the delivery of drugs due to its unique characteristics such as swelling and adsorption. Salahuddin et al. has recently reported 5-phenyl-1,3,4-oxadiazole-2-thiol/polyamide-MMT microbicide nanocomposites in the field of drug delivery [77]. These MMTs with anionic polymers like alginate form a unique polymer silicate material that provides a superior capability of incorporating drug molecules within the structure. Clay minerals played the role of multi-functional cross-linkers in nanocomposite hydrogel with improved mechanical properties. Further, the in vitro studies supported the use of polyamide-MMT in the sustained release of 1,2,3-oxadiazoles via oral administration. 

In 2017, Yavvari et al. designed synthetic polyamide polymers with ionic hydrophobic counterparts to target Mycobacterium tuberculosis [78]. Synthetic antimicrobial polymers (SAMPs) derived from biodegradable, water-soluble polymers such as polyaspartic acid showed hydrophobicity and antimicrobial activity against both Gram (+) and (−) bacteria. With an increase in the hydrophobicity of SAMPs, their activity to permeabilize mycobacterial diminished membranes. However, a lower hydrophobicity offered effective biofilm disruption indicating higher perfusion into the biofilm matrix. Menezes et al. have reported hesperetin-loaded lipid-core nanocapsules (LNCs) in polyamide gel to formulate a medical textile formulation for topical delivery against chronic venous insufficiency. The oily core of these nontoxic polymeric nanocapsules was formed by an organogel comprising of sorbitan monostearate and capric/caprylic triglyceride. The chemical composition of the core of the nanocapsules controlled drug penetration into various tissues. After topical application, the drug was released in response to skin stimuli such as sweat. This study showed the significantly higher extraction of drugs through LNCs as compared to the conventional one. Further, LNCs showed increased retention in the stratum corneum and thus provided controlled drug release [79]. Polyamides are also explored for sutures in cutaneous surgery and clinical treatments of wounds by Sun et al. [80]. The formulation of polyamide 6,6 with N-acetylcysteine was preferred for the sustained release in wound healing. The stability of formulation was further enhanced due to the multi-layered structure of polyamides. These scaffolds with collagen provided a better water absorption capacity, biocompatibility and sustained release profile than polyamide nanoparticles due to the higher resistance of polyamides to solvent and body fluids to influence wound healing.

## 3. Poly(Amino Acid)-Based Micelles Undergoing Clinical Trials

**Figure 5:**
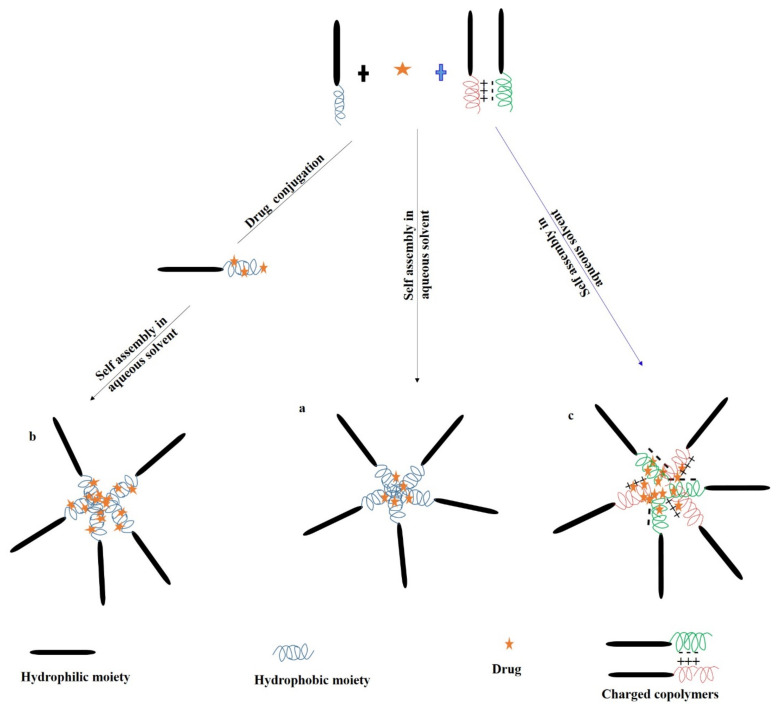
Common types of polymeric micelles: (**a**) block copolymer micelle; (**b**) drug-conjugated block copolymer micelle; and (**c**) block ionomer complex micelle. Modified from [82].

**Figure 6:**
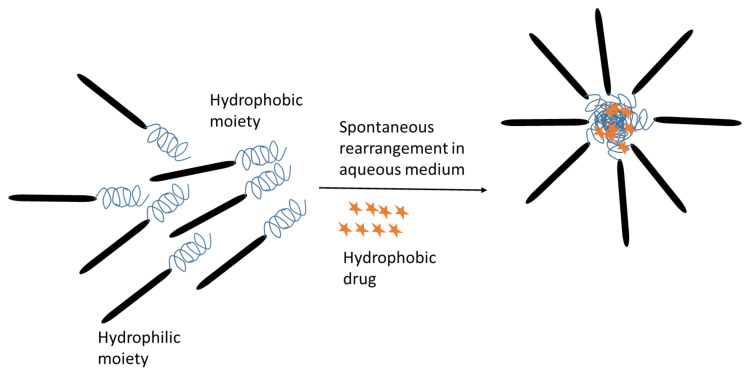
Spontaneous formation of micelles from amphiphilic molecules in aqueous media, while entrapping hydrophobic drugs.

Poly(amino acid) polymers are extensively investigated in the delivery of water-insoluble drugs by entrapping them in the inner core of polymeric micelles. Polymeric micelles are self-assembling nanosized particles consisting of a hydrophobic core/hydrophilic coat or shell. Amphiphilic copolymers have received tremendous commercial attention as micelle-forming compounds. Micellar systems have a particle size ranging between 5–100 nm. The dispersed systems consist of a dispersed phase which is particulate in nature distributed within a dispersion medium which constitutes the continuous phase [81]. There are three types of micellar drug delivery systems based on linear block copolymers (Figure 5). They are: (1) block copolymer micelle, (2) drug-conjugated block copolymer micelle and (3) block monomer complex micelle. The illustrative scheme of micelle formation from an amphiphillic molecule, loaded with a poorly soluble drug and different mechanisms to alter micelle for improving its properties as a drug carrier is shown in Figure 6.

Polymeric micelles due to their smaller size and enhanced properties tend to show the increased accumulation of drugs in tumor tissues utilizing the enhanced permeability and retention (EPR), and incorporate a variety of drugs into the inner core either by physical entrapment or by chemical conjugation with relatively high stability. The nanosize of the micelles prevents their entry through normal vessel walls and reduces the incidence of side effects of the cytotoxic drugs while decreasing the volume of distribution [83]. The EPR effect that governs the passive targeting of drugs and therapeutics to the target tissues, organs or cells is attributed to the pathophysiological characteristics of solid tumor tissues such as: (a) hypervascularity, (b) vascular permeability factors secreted within cancer tissue stimulates extravasation and (c) the absence of effective lymphatic drainage in tumors due to which the macromolecules accumulated in solid tumor tissues are not efficiently cleared. In the past decade or so, several techniques of drug delivery have been investigated maximally using the EPR such as the modification of drug structures and development of drug carriers for targeted drug delivery in the delivery of mainly oncological drugs. The core of polymeric micelles is generally made up of biodegradable polyesters such as poly(D,L-lactide), poly(D,L-lactide-co-glycolide) and poly(ε-caprolactone). PEG-b-poly(amino acids) copolymers are also a popular choice due to the flexibility in selecting the amino acids of choice and adaptable side chain modification for optimizing the properties of micelles [84,85]. Polymeric micelles made of poly(amino acid) polymers that are in the clinical trials are discussed thoroughly in the following section. A detailed discussion on the method of preparation, characterization and pharmacokinetic/ pharmacodynamic evaluation has been included.

### 3.1. NK 105, Paclitaxel Incorporating Micelles

**Figure 7:**
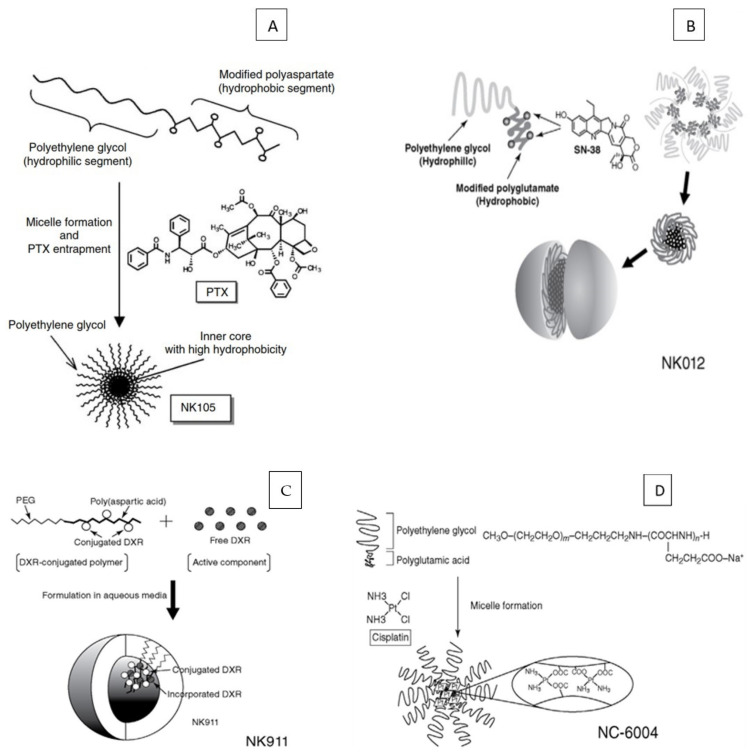
Schematic presentation of poly(amino acid)s-based micelles systems: (**A**), NK105, (**B**) NK012, (**C**) NK911, (**D**) NC-6004. (Reproduced with permission from Refs. [92,93]).

Paclitaxel is one of the most potent and useful anticancer drugs available in the treatment of a variety of cancers such as ovarian, breast and lung cancers [86,87]. However, paclitaxel treatment is also known for its severe unwanted effects such as neutropenia, peripheral sensory neuropathy, anaphylaxis and other hypersensitivity reactions. It is also shown to exhibit allergic reactions in 2–4% of the patients. These adverse reactions can be attributed to the use of the typical formulation composition of paclitaxel (a mixture of Cremophor EL and ethanol as co-solvents) which is used to solubilize the drug [88,89]. Several attempts have been made to minimize the severity of paclitaxel side effects during the treatment. For example, neutropenia can be minimized or completely prevented by the administration of a granulocyte colony stimulating factor. However, attempts to prevent/reduce nerve damage (which is primarily caused due to paclitaxel itself) have shown no beneficial results [90,91]. NK 105 is a promising micellar nanocarrier drug delivery system where in paclitaxel was incorporated into polymeric micelle, developed by Nanocarrier^®^ (Chiba, Japan) and is presently at the stage of clinical trials by Nippon Kayaku. The polymeric system involved in designing NK 105 consists of poly(ethylene glycol)-poly(aspartic acid) modified with the use of 4-phenyl-1-butanol in order to enhance the hydrophobicity (Figure 7A) [92]. 

#### 3.1.1. Method of Preparation

The block copolymers consisting of PEG and polyaspartate were used as building blocks [94,95,96]. Paclitaxel was incorporated into polymeric micelles by the physical entrapment method. The formation of micelles was due to hydrophobic interactions between paclitaxel and the block copolymer polyaspartate chain. Next, 4-phenyl-1-butanol was employed to increase the hydrophobicity of the polyaspartate block (by chemical modification). Nearly half of the -COOH groups present on the polyaspartate were esterified with 4-phenyl-1-butanol using 1,3-diisopropylcarbodiimide as a condensing agent. NK 105 was prepared by self-assembly of NK105 and paclitaxel. The nanoparticles with an average diameter of 85 nm (ranging from 20 to 430 nm) were formed.

#### 3.1.2. Pharmacokinetics and Pharmacodynamics of NK105

The NK105 paclitaxel micellar nanoparticles were evaluated for their pharmacokinetic and pharmacodynamic performance in animal models. For the pharmacokinetic evaluation, colon-26 bearing CDF1 mice were selected. They were then injected (I.V.) with PTX 50 or 100 mg/kg, or of NK105 at a comparable dose of PTX. The in vivo pharmacokinetic parameters were assessed in plasma as well as target tumor tissues. As shown in Table 3, it was found that NK105 polymeric micellar nanoparticles were slowly cleared from the plasma compared to paclitaxel, with the former being detected up to 72 h in plasma after injection whereas the latter was not detected after 24 h [97]. 

#### 3.1.3. In Vivo Pharmacodynamic and Toxicity Study of NK105

*Anti-tumor activity*: The anti-tumor activity studies performed in BALB/c mice bearing s.c.HT-29 colon cancer tumors indicated that both paclitaxel and NK105 decreased the rate of tumor growth after injection. However, NK105 demonstrated better anti-tumor activity than paclitaxel; therefore, the activity obtained at dose levels of 25 mg/kg of NK105 was comparable to 100 mg/kg of paclitaxel alone. NK105 showed a dose-dependent tumor suppression effect. 

*Neurotoxicity studies:* Paclitaxel administration often results in cumulative peripheral neurotoxicity which often manifests as numbness and or parasthesia of the extremities. This ultimately results in the swelling of axoms, vascular degeneration and de-myelination. Electrophysical and morphological experimental methods for assessing the neurotoxicity of NK105 and paclitaxel showed that both were similar in amplitude of the caudal sensory nerve activity potential while the amplitude of SNAP (sensory nerve action potential) was considerably higher for NK105 compared to paclitaxel. Histopathological studies of sciatic nerves displayed only slight degenerative myelineated fibers compared to paclitaxel [97].

*Radiosensitizing effect:* Radiation therapy, a commonly employed method in the treatment of tumors, is associated with severe toxicity effects on lungs (breast and lung cancer) resulting in lung fibrosis or even mortality. In order to overcome these adverse effects, NK105 micellar nanoparticles were proposed as a better alternative to paclitaxel plus radiation therapy. NK105 plus radiation showed improved cell cycle arrest at G2/M phase compared to paclitaxel. The combination of NK105 plus radiation showed a decreased rate of tumor growth in mice of radiation alone and paclitaxel plus radiation [98].

#### 3.1.4. Clinical Studies

An extensive phase I clinical study was conducted to investigate the maximum tolerated dose and dose limiting toxicities. In addition, the recommended dose for phase II clinical trials and the pharmacokinetic profile of NK105 were also investigated [99]. NK 105 was intravenously administered as an infusion every 3 h without the pre-medication of anti-allergic therapy. The study was initiated with the dose of NK105 equivalent to 10 mg of paclitaxel and accelerated titration method was followed for dose escalation in the studies. The study involved 17 patients treated at doses ranging from 10 mg to 180 mg. Various types of tumors treated include pancreatic bile duct, gastric and colon. The histopathological toxicity was assessed by grading the severity of neutropenia, a commonly observed toxicity of paclitaxel. NK105 treated patients showed an improved side effect profile compared to paclitaxel. In addition, the paclitaxel-induced allergic reactions were not observed in NK105 treated patients. The C_max_ and AUC plasma profile of NK 105 displayed dose-dependent properties. The plasma AUC of NK105 at a treatment dose of 180 mg/m^2^ showed a 30-fold increase compared to paclitaxel formulation. In addition, NK105 infusion tri-weekly for one hour was well tolerated and feasible in patients of pancreatic cancer, showing anti-tumor activity. In conclusion, phase I clinical trials for NK105 in patients having different tumors showed an enhanced pharmacological response along with reduced toxicity or adverse effects [99]. A phase II study was carried out to assess the safety and efficacy of NK105 in advanced gastric cancer patients. NK105 was dosed at 150 mg/m^2^ by a 30-min IV infusion every 3 weeks. The median progression-free survival was 3.0 months, while the median time to treatment failure and the median overall survival was 2.8 months and 14.4 months, respectively. Only mild drug-related toxicity was observed with no treatment-related deaths. This phase II study showed the modest activity and tolerability of NK105 [100]. More recently, a multi-national, open-label, randomized, parallel, phase III non-inferiority trial was conducted in 436 patients to compare the efficacy and safety of NK105 and PTX in metastatic/recurrent breast cancer with the primary endpoint of progression-free survival (non-inferiority margin of 1.215). Patients were divided into two groups in a random manner and received either PTX (80 mg/m^2^) or NK105 (65 mg/m^2^) on days 1, 8 and 15 of a 28-day cycle. This study showed a better peripheral sensory neuropathy toxicity profile of NK105 than PTX, the primary endpoint was not met [101]. Further studies are needed to re-evaluate the efficacy of NK105.

#### 3.1.5. Other Similar Products in the Pipeline 

In 2016, Nippon Kayaku obtained orphan drug status for NK012, a polymeric micelle designed by covalently linking SN-38 to the block copolymer of PEG-block-poly(L-glutamic acid), for treating small cell lung cancer (Figure 7B). NK012 releases SN-38 via hydrolysis and does not require enzymes for metabolism. NK012 has completed phase II clinical trials for relapsed small cell lung cancer and triple-negative breast cancer [102]. More studies are needed to decide the ideal dose of NK012 to increase safety and efficacy. Additionally, further investigations on various combination therapies with NK012 and other anticancer drugs are underway [103]. NK911 contains doxorubicin linked to the poly(Asp) chain of PEG-b-P(Asp) block copolymer with an amide linkage along with physically entrapped doxorubicin (Figure 7C). The phase I studies of NK911 were conducted in 23 patients in 2001. The results indicated that the NK911 injection was tolerated well and non-haematological toxicities such as nausea, vomiting and anorexia were reported. The haematological toxicity data showed neutropenia (grade 3 and 4) at dosing of 50 mg·m^−2^ and the toxicity increased at a higher dose of 67 mg·m^−2^. Therefore, 50 mg·m^−2^ was considered as the suggested dose for phase II clinical trials for the assessment of efficacy along with toxicity profiles. However, the results of the phase II clinical trials are still pending [104].

### 3.2. NC-6004, Cisplatin-Incorporating Micellar Nanoparticle

Cisplatin [cis-dichlorodiammineplatinum (II)], is an important member of platinum-containing anticancer drugs and is used in treating various cancers, including malignancies of lungs, GIT and genitourinary tracts [105,106,107]. These platinum complexes cause programmed cell death through binding and cross-linking of DNA. The administration of cisplatin in conventional formulations is often associated with unwanted effects such as nephrotoxicity and neurotoxicity. Newer platinum analogues have also been developed and investigated such as carboplatin and oxaliplatin [108], which upon evaluation in various malignancies, have become standard drugs for ovarian cancer [109] and colon cancer [110]. Cisplatin is also considered as a part of the standard regimen for treating lung cancer, stomach cancer and testicular cancer. Thus, considering the importance of cisplatin as an anticancer drug, there is a need for improving its pharmacological profile with an improved adverse effect profile. NC-6004 is a nanocarrier system currently in phase II clinical trials. NC-6004 is designed using micellar technology for the sustained release of cisplatin in treating various tumors [111].

#### 3.2.1. Preparation of NC-6004 Micellar Complex

The micellar formulation of NC-6004 is made up of polyethylene glycol (PEG) in the corona (outer shell) of micelles and the inner core comprises of a coordinate complex of poly(glutamic acid) (P(Glu)) and cisplatin (Figure 7D). The ligand substitution of platinum(II) atom from -Cl^−^ to -COO^−^ in the side chain of P(Glu) is the key driving force in the formation of the cisplatin polymeric micellar complex. The polymeric micellar complex has a particle size of 30 nm. The complex was non-dissociating upon dilution and the critical micellar concentration value was found to be 5 × 10^−7^ [47]. The drug release from NC-6004 occurred in a sustained manner in physiological saline. This was attributed to an inverse ligand substitution of Pt (II) atom from a glutamate residue of PEG-P(Glu) to chloride [46]. Furthermore, the affinity between the Pt (II) and glutamate residue is critically optimal for anti-tumor activity and its toxicity due to the enhanced stability of the complex in vitro and in vivo [112]. The complex has also been shown to possess pH dependent drug release (increased drug release when pH was decreased from 7.4 to 5.2), which is critical therapeutically as the enhanced drug release from NC-6004 in the endosomal lysosomal system upon transportation into the target tissue as micellar complex form. The preparation and characterization of NC-6004 has been depicted in Figure 7 [46,47].

#### 3.2.2. Pharmacokinetics and Pharmacodynamics

The pharmacokinetic evaluation of NC-6004 in animals showed that NC-6004 was retained in the blood for a long duration as compared to cisplatin [46]. The C_max_ and AUC_0__–t_ values were found to be higher for NC-6004 than cisplatin alone. The results of the pharmacokinetic studies are shown in the Table 4. The concentration-time profile analysis of cisplatin or NC-6004 showed that both have high initial concentrations attained within 1 h of intravenous administration. The NC-6004 higher tissue concentrations of platinum (Pt) were observed in the liver and spleen at 24 h and 48 h post-injection, respectively. The concentrations of Pt at 48 h post-injection were 4.6 and 24.4 folds higher for NC-6004 than cisplatin in the liver and spleen, respectively [46]. The peak tumor tissue concentration from cisplatin was found to be at 10 min post-intravenous administration while that for NC-6004 was highest at 48 h post-injection, with C_max_ 2.5 folds higher for NC-6004 compared to cisplatin.

#### 3.2.3. In Vivo Anti-Tumor Activity

The anti-tumor activity studies performed in BALB/c nude mice implanted with MKN-45 cell line indicated that the rate of tumor growth was decreased for both cisplatin and NC-6004. The NC-6004 administration equivalent to cisplatin had no significant difference in the rate of tumor growth. The time course of rate of change in body weight for NC-6004 showed no change, while cisplatin showed a significant decrease in body weight, which indicated the superiority of NC-6004 in treating tumors. The in vitro cytotoxicity studies of NC-6004 performed on human tumor cells form bladder, colon, lung, gastric and breast cancer tissues, suggested that the IC_50_ were about 6–15 fold higher than those for cisplatin. The decreased cytotoxicity of the micellar complex was predominately due to the sustained release of cisplatin from the complex [46].

#### 3.2.4. Nephrotoxicity of Cisplatin and NC-6004

The administration of cisplatin at 10 mg/kg levels in rats resulted in deaths of about 25% of animals. However, no deaths occurred in the case of cisplatin polymeric micelle, NC-6004. The administration of NC-6004 showed no histopathological change on the tubular epithelium cell-lining of kidneys upon light microscopy studies, whereas cisplatin showed tubular dilation with flattening of tubular epithelium cell-lining of the kidney. The neurotoxicity assessment studies of NC-6004 showed no delay in sensory conduction velocities (SNCV) as compared with cisplatin. The pharmacokinetic studies of NC-6004 compared to cisplatin showed that the NC-6004 micellar complex was circulating in the plasma for a prolonged period of time [46,93], with AUC and C_max_ were 65 and 8 fold higher than that of cisplatin. The disposition studies revealed a higher distribution in the liver and spleen (RES system) and were found to be eliminated via kidneys. Improved therapeutic attributes in terms of better tissue distribution and accumulation at target tissues, and reduced toxicity profiles all resulted in an increased interest to develop into a clinically viable formulation, thus facilitating the compound to enter clinical studies.

#### 3.2.5. Clinical Studies of NC-6004

Phase I clinical trials of NC-6004 involving intravenous administration of the drug within one hour every 3 weeks (dose range: 10–120 mg/m^2^) showed that the treatment is well-tolerated with minimum nephrotoxicity. However, NC-6004 showed frequent hypersensitivity reactions than cisplatin at all dose levels. From these studies the maximum-tolerated dose and recommended dose for phase II trials were fixed to 120 mg/m^2^ and 90 mg/m^2^, respectively. The PK studies showed a longer circulation time in the blood for NC-6004 with a linear pharmacokinetic profile. This shows an inferior cisplatin-related toxicity and superior anti-tumor activity of NC-6004 compared to cisplatin [113]. Recently, phase IIa was completed by enrolling patients in five sites in Europe by a traditional 3 + 3 design. NC-6004 was administered intravenously at 90, 105, 120 or 135 mg/m^2^ for over 1 h on day 1. Pembrolizumab (200 mg) was administered intravenously over 30 min on day 1 every 3 weeks. The maximum-tolerated dose and recommended phase 2 dose was reported to be 135 mg/m^2^, which is more than the regular cisplatin doses in the head and neck squamous cell carcinoma treatment. The neurotoxicity or nephrotoxicity of NC-6004 was significantly lower than the conventional cisplatin regimen with a decent safety profile [114].

#### 3.2.6. Other Similar Products in the Pipeline

NC-4016 is oxaliplatin-loaded in PEG-b-poly(l-glutamic acid) micelles with a diameter of 40 nm and drug of 32% (*w*/*w*). In the aqueous medium, NC-4016 is assembled as a result of the complex formation between -COOH groups in P(Glu) and platinum in oxaliplatin. In 2017, a dose-escalation phase I of NC-4016 in 34 patients suffering from advanced solid tumors or lymphoma was completed. Varying doses of 15–80 mg/m^2^ every 3 weeks were administered; however, no results have been disclosed so far [115,116]. NC-6300 is epirubicin covalently linked to the PEG12000-polyaspartate block copolymer with pH sensitive hydrazone bond. NC-6300 micelles have a diameter of 40–80 nm and releases approximately 80% of epirubicin at pH 3.0 within 1 h. However, at neutral pH only about 20% of epirubicin was released within 48 h [116]. A phase I study of NC-6300 was conducted in 19 patients suffering from advanced/recurrent solid tumors. The results indicated that NC-6300 had less toxicity and was more well-tolerated than the conventional epirubicin formulation [117]. The recommended dose of NC-6300 for phase II studies was suggested to be 170 mg/m^2^. The phase II study of NC-6300 is underway to assess its tolerability and anti-tumor activity in soft tissue sarcoma patients.

## 4. Challenges and Future Directions

The contributions of polymer science to the evolving field of drug delivery have been very significant, and there are many examinations underway. Poly(amino acid) polymers have been around for quite some time in drug delivery due to their similarity to proteins and highly biocompatible degradation products. Though poly(amino acid) polymers are hydrophilic, they are easy to alter by linking different functionalities (-NH_2_, -OH and -SH) in the polymer backbone [118]. The challenges faced by poly(amino acid) polymers include poor mechanical characteristics, the tendency to produce immunogenicity due to amide bonds and exhibit charge-induced toxicity. The immunogenicity and poor mechanical strength of poly(amino acid) polymers led to the development of pseudo poly(amino acids), which are obtained by linking other synthetic polymers via non-amide bonds like esters, carbonates and imino-carbonates [119]. For instance, tyrosine-derived poly(amino acids) belong to the category of pseudo poly(amino acids) with good physical and mechanical properties. It should be noted that the drug delivery systems such as polymeric micelles and dendrimers designed using poly(amino acid) polymers are still evolving and more research is needed for such systems to become viable alternatives.

The major hurdle that delays the clinical translation of polymeric micelles is their inability to retain integrity in the blood following intravenous injection. Polymeric micelles within the blood tend to remain either in the assembled state or as mesostructures comprising of macromolecule unimers [120]. The pH and salt changes and interaction with proteins generally destabilize the micellar structure after dilution in the blood, which results in the premature release of the payload release before reaching the target tissue. The stability of polymeric micelles depends on kinetic and thermodynamic aspects. Polymeric micelles with CMC value < 1–5 mg/L are generally more stable [121]. Considering the blood volume to be 6 L, a minimum polymer dose of 6–30 mg is required for an average adult based on the CMC value of 1–5 mg/L [122]. The in vivo fate of polymeric micelles injected into blood depends on their interactions with serum proteins such as albumin, globulins, apolipoprotein, fibrinogen and immunoglobulin-G. For instance, the COO^−^ form of camptothecin is in balance with its active lactone form in the bulk phase. When human serum albumin interacts with the COO^−^ form of camptothecin, the equilibrium was moved to its HSA-binding carboxylate form leading to leakage of camptothecin from poly(ethylene glycol)-poly(benzyl aspartate-69) block copolymer micelles [123]. Studies indicate the proteins adsorb onto the surface of nanocarriers such as polymeric micelles within a few minutes of exposure, especially when the surface is hydrophobic or charged. The adsorption of plasma proteins on drug-loaded micelles may lead to dissociation or aggregation. For example, PEG-b-poly(γ-propargyl-glutamate) copolymer was linked to various 4-azido-butylbenzenes bearing hydrophobic groups for preparing a series of amphiphilic block copolymers. Paclitaxel was loaded into these micelles and incubated with fetal bovine serum (20%) at 37 °C. The results indicated that paclitaxel-loaded micelles of copolymers P2–P6 rapidly dissociated in the presence of serum protein [124].

Despite the attractive properties of polymeric micelles in drug delivery, drug entrapment via simple physical entrapment may not result in sufficient stability of drug-loaded micelles in vivo. The presence of multiple interactions such as the hydrophobic/electrostatic interaction may provide better in vivo stability to drug-loaded micelles. Various other strategies such as covalent cross-linking of the micellar core and coating of drug-loaded micelles have been reported in the literature to overcome challenges faced by polymeric micelles. Covalent core cross-linking of the micelles’ core has garnered considerable attention in the production of stable polymeric micelles for drug delivery applications. In a study, the ionic cores of phenylalanine-linked poly(ethylene glycol)-b-poly(L-glutamic acid) in the presence of calcium ions were chemically cross-linked with cystamine. This study demonstrated that hydrophobic moieties in the ionic cross-linked cores of nanogels greatly affected the release of doxorubicin at simulated lysosomal acidic pH and showed an improved anti-tumor activity in an ovarian tumor xenograft mouse model [125]. The other strategy of coating polymeric micelles with polyanionic agents helped in the specific targeting and accumulation of micelles into liver sinusoidal endothelial cells. Anionic hyaluronic acid-coated micelles of poly(l-lysine)-block-poly(l-lactide) (PLys-b-PLLA) AB diblock copolymers showed significantly higher stability in the aqueous solution. Moreover, hyaluronic acid-coated micelles were taken up only into liver sinusoidal endothelial cells, while heparin-coated micelles and carboxymethyl-dextran-coated micelles were accumulated into both Kupffer cells and liver sinusoidal endothelial cells [126]. Overall, poly(amino acid) polymers have a promising role in drug delivery, especially in chemotherapy. However, further studies in humans are needed to better understand their advantages and drawbacks.

## 5. Conclusions

Drugs are normally administrated orally, as tablets, capsules, powders or liquid forms. As time has advanced the need for having delivery systems that could sustain the drug release at the target site has become apparent. During the last couple of decades, polymer science has played a vital role in the design of novel drug delivery systems. Most polymers do not have the natural ability to function optimally as drug delivery devices and this can be achieved by innovative modifications of their physicochemical and physicomechanical properties during synthesis. From the above discussion it is quite obvious that polyamides or poly(amino acids) play a prominent role in the delivery of drugs. Poly(amino acids) are simple and highly adaptable polymers with protein-like properties. They are ideal for drug/nucleic acid delivery and provide suitable drug targeting, release characteristics and bypass multidrug resistance (MDR) factors upon chemical modifications. Several polyamide-based delivery systems were designed to augment the therapeutic properties of drugs and make them more safe and effective in disease treatment. The flexible nature of poly(amino acids) helps modify micellar structures readily to incorporate new features based on the information revealed from clinical trials. Such modifications would result in the design of novel formulations with better performance. Since the last two decades, polyamides have been widely investigated for the delivery of bioactive agents; however, their extensive use is limited by poor control over the release rate of the drug, antigenic potentials and the requirement of enzymes for degradation in the body. With several poly(amino acid)-based micelles in clinical trials, it is very likely that poly(amino acid)s will play a growing role as high performance materials in the medical field.

## Figures and Tables

**Table 1:** Classification of polymers employed in drug delivery.

Classification	Polymer
Natural Polymers	
Protein–based polymers	Collagen, albumin, gelatin
Polysaccharides	Agarose, alginate, carrageenan, hyaluronic acid, dextran, chitosan, cyclodextrins
Synthetic polymers	
Biodegradable	
Polyesters	Poly(lactic acid), poly(glycolic acid), poly(hydroxy butyrate), poly(ε-caprolactone), poly(β-malic acid), poly(dioxanes)
Polyanhydrides	Poly(sebacic acid), poly(adipic acid), poly(terphthalic acid) and various copolymers
Polyamides	Poly(imino carbonates), polyamino acids
Phosphorus-based polymers	Polyphosphates, polyphosphonates, polyphosphazenes
Others	Poly(cyano acrylates), polyurethanes, polyortho esters, polydihydropyrans, polyacetals
Non-biodegradable polymers	
Acrylic polymers	Polymethacrylates, poly(methyl methacrylate), poly hydro(ethyl methacrylate)
Cellulose derivatives	Carboxymethyl cellulose, ethyl cellulose, cellulose acetate, cellulose acetate propionate, hydroxyl propyl methyl cellulose
Silicones	Polydimethyl siloxane, colloidal silica
Others	Polyvinyl pyrrolidone, ethyl vinyl acetate, poloxamers, poloxamines

**Table 2:** Structure of polyamide/poly(amino acid) polymers in drug delivery.

Polymer	Structure	Applications
Poly(lysine)	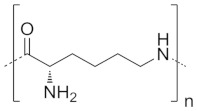	Used as an antimicrobial agent in food and pharmaceutical industry. It is recognized as a GRAS material by FDA in USA. Used in manufacturing of sanitation napkin and diapers because of its good absorption of liquids. Used as a carrier for pharmaceutical molecules and cosmetics.
Poly(glutamic acid)	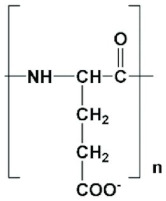	Cosmetics industry: used in skin, body and hair care products. Food industry: used as a food additive. Construction industry: used as an additive in paints and concrete products. Pharmaceutical industry: used in tablet coating and controlled delivery of drugs as polymer conjugates.
Poly(α-L-aspartic acid)	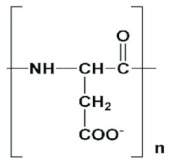	Pharmaceutical preparations (drug delivery systems, percipients in tablets). Used as a fertilizer, scale inhibitor, corrosion inhibitor, pesticide and superabsorbent material. Used as a cementing agent or sand fixing agent due to its ability to aggregate sand particles.
Polyamidoamine (G1)	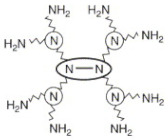	Used as carriers for targeted drug and gene delivery and bio-imaging.

**Table 3:** Pharmacokinetic parameters for the plasma and tumor concentrations of paclitaxel after single intravenous administration of NK 105 and paclitaxel to colon-26 bearing CDF1 mice (reproduced with permission from Ref. [92]). i.v. = intravenous; C_5 min_= plasma concentration at 5 min; t_1/2z_ = half-life at the terminal phase; AUC = area under the curve; CL_tot_ = total body clearance; V_ss_ = volume of distribution at steady state; T_max_= time of maximum concentration; PTX = paclitaxel. Parameters were calculated from the mean value of three or two mice by noncompartmental analysis.

Treatment	Dose (mg/kg)	C_5 min_ (µg/mL)	t_1/2 z_ (h)	AUC_0-t_ (µg·h/mL)	AUC_0-inf._ (µg·h/mL)	Cl_tot_ (mL/h·kg)	V_ss_ (L/kg)
Plasma PTX	50	59.32	0.98	90.2 ^a^	91.3	547.6	684.6
PTX	100	157.67	1.84	309.0 ^b^	309.0	323.6	812.2
NK105	50	1157.03	5.99	7860.9 ^c^	7862.3	6.4	46.4
NK105	100	1812.37	6.82	15,565.7 ^c^	15,573.6	6.4	54.8
**Treatment**	**Dose (mg/kg)**	**C_max_ (µg/mL)**	**T_man_ (h)**	**t_1/2 z_ (h)**	**AUC_0-t_ (µg·h/mL)**	**AUC_0-inf._ (µg·h/mL)**	
Tumor PTX	50	12.50	2.0	7.02	120.8 ^b^	133.0	
PTX	100	28.57	0.5	8.06	330.4 ^c^	331.0	
NK105	50	42.45	24.0	35.07	2360.1 ^c^	3192.0	
NK105	100	71.09	6.0	73.66	3884.9 ^c^	7964.5	

^a^ AUC_0__–6 h_, ^b^ AUC_0__–24 h_, ^c^ AUC_0__–72 h_.

**Table 4:** Pharmacokinetic parameter estimates for cisplatin (CDDP) and NC-6004 in rats (reproduced with permission from Ref. [46]).

Compound	Rat	T_max_ ^a^ (h)	C_max_ ^a^ (µg/mL)	t_1/2 z_ (h)	AUC_0-t_ (µg·h/mL)	AUC_0-inf._ (µg·h/mL)	CL_tot_ (mL/h·kg)	MRT_0-inf._ (h)	V_ss_ (L/kg)
CDDP	Mean s.d.	0.083	11.67 0.57	34.50 16.14	20.47 2.25	75.73 26.13	70.67 20.34	46.57 22.38	3.00 0.61
NC-6004	Mean s.d.	0.50	89.90 4.29	6.43 0.55	1325.90 77.85	1335.47 75.99	3.77 0.21	10.67 0.15	0.04 0.0023

The pharmacokinetic parameters were calculated after fitting to a noncompartment model using WinNonlin program. ^a^ For CDDP group, T_max_ represents time of maximum concentration.

## Data Availability

The data presented in this study are available on request from the corresponding author.
